# First record of the tropical aphid *Schoutedeniaralumensis* (Hemiptera, Aphididae) from Cambodia, with a re-description of the oviparous females and DNA barcoding

**DOI:** 10.3897/BDJ.13.e159374

**Published:** 2025-08-04

**Authors:** Hwalran Choi, Colin Favret, Seunghwan Lee

**Affiliations:** 1 Seoul National University, Seoul, Republic of Korea Seoul National University Seoul Republic of Korea; 2 University of Montreal, Montreal, Canada University of Montreal Montreal Canada

**Keywords:** aphid, COI, Cambodia, *Phyllanthus*, *Schoutedeniaralumensis*, oviparous females

## Abstract

**Background:**

*Schoutedenia* Rübsaamen, 1905 (Hemiptera, Aphididae, Greenideinae) is a small aphid genus associated with woody Euphorbiaceae and Phyllanthaceae. Of its two recognised species, *Schoutedeniaralumensis* Rübsaamen, 1905 is widely distributed across Southeast Asia, India, Africa and along the eastern coast of Australia. Taxonomic difficulties arise from subtle morphological differences and an unusual life cycle in which all morphs may occur simultaneously. Moreover, the oviparous female remains inadequately described, limiting reliable identification and comparative analyses.

**New information:**

*Schoutedeniaralumensis* is newly recorded from Cambodia on *Phyllanthus* sp. (Phyllanthaceae). The poorly-known morphology of oviparous females is re-described with live photographs, biometric measurements and photomicrographic illustrations. Additionally, DNA barcoding, based on mitochondrial cytochrome c oxidase (COI) sequences, was performed on the Cambodian specimen and compared with available sequences, including one of *S.emblica*. Additionally, we propose the synonymisation of *S.emblica* under *S.ralumensis* as a conspecific variant. These findings expand the known distribution of *S.ralumensis* and contribute to a better understanding of aphid diversity in Cambodia.

## Introduction

The genus *Schoutedenia* Rübsaamen, 1905 is an aphid group belonging to the subfamily Greenideinae (Hemiptera, Aphididae). It was revised by Remaudière (1988) and Remaudière (1990), who recognised two valid species: *S.ralumensis* Rübsaamen, 1905 and *S.emblica* (Patel and Kulkarni, 1952). These aphids are considered part of an ancient Gondwanan lineage and are predominantly associated with woody hosts in the families Euphorbiaceae and Phyllanthaceae. They exhibit monoecious and holocyclic life cycles, often with subtle morphological differences (Favret and Aphid Taxon Community, eds 2025).

Amongst the two species, *Schoutedeniaralumensis* is the more widely distributed. It is found across a broad geographic range including Southeast Asia (e.g. Bangladesh, Laos, Thailand), the Indian subcontinent, parts of Africa and the east coast of Australia (Ghosh and Agarwala 1993, Chen et al. 2020, Favret and Aphid Taxon Community, eds 2025). It colonises multiple genera within Euphorbiaceae and Phyllanthaceae, such as *Breynia*, *Bridelia*, *Flueggea*, *Glochidion* and *Phyllanthus*, displaying a relatively wide host range. In contrast, *S.emblica* is restricted to *Phyllanthusemblica* L. as its host and has a more restricted distribution, confined to India, Pakistan, Nepal, Thailand, China and the western Himalayas (Bhattacharya and Dey 2001, Zhang et al. 2006).

Species identification within the genus has historically been problematic due to subtle morphological differences, particularly in traits, such as the ratio of the ultimate rostral segment (URS) to the processus terminalis (PT) and the relative proportions of antennal segments. Although *S.emblica* was once synonymised with *S.ralumensis* by Remaudière (1988), it was later re-instated (Remaudière 1990) and its status remains contentious. Molecular data have provided additional insight: a Neighbour–Joining tree, based on mitochondrial cytochrome c oxidase I (COI) sequences by Liu et al. (2013), placed *S.emblica* within *S.ralumensis*, suggesting possible conspecificity or deep intraspecific divergence within *S.ralumensis*.

Here, we report a new occurrence of *Schoutedeniaralumensis* Rübsaamen, 1905 from Cambodia, expanding its known geographic distribution. We also provide the first detailed morphological description of the oviparous female morph, which was previously poorly characterised. Earlier taxonomic accounts, such as Ghosh and Agarwala (1993), focused on the apterous and alate viviparous forms. They provided measurements, illustrations and host plant information. However, descriptions of other morphs — especially the oviparous form — were lacking.

In this study, we offer a full morphological re-description of the oviparous female, based on specimens collected on *Phyllanthus* sp. (Phyllanthaceae). Importantly, we provide the first ecological photograph of a live oviparous female during oviposition, together with its COI barcode data. This integrated documentation improves species identification and understanding of the life cycle of *S.ralumensis*. Additionally, we contribute to global aphid datasets by adding a new mitochondrial COI sequence from a Cambodian specimen, supporting efforts to resolve taxonomic ambiguities in *Schoutedenia*.

## Materials and methods

### Taxon sampling and morphological identification

Fresh aphid samples were collected in Koh Kong Forest Station, Cambodia in 2011. Approximately 40 individuals from a colony were preserved in 90% ethanol and 12 oviparous females were mounted on glass slides in Canada balsam, following Martin (1983)the Blackman and Eastop (2000) method. Species identification was based on the morphological characteristics of apterous viviparous females, following the keys provided by Ghosh and Agarwala (1993) and Favret and Aphid Taxon Community, eds (2025). Illustration for one species was taken by digital camera (14.2 Color Mosaic, Diagnostic Instruments, Sterling Heights, MI, USA) attached to the microscope (DM 400B, Leica Microsystems, Wetzlar, Germany) at a resolution of 600 dpi. Measurements for each specimen are taken from the digital images by Image Laboratory v.2.2.4.0 software (MCM Design Ltd, Hillerod, Denmark). Specimens collected during this study are deposited in the College for Agriculture and Life Sciences, Seoul National University (CALS SNU, Korea).

Abbreviations used for description are as follows: Ant., antennae; Ant.I, Ant.II, Ant.III, Ant.IV, Base, PT and Ant.IIIBD, antennal segments I, II, III, IV, basal part of last antennal segment, processus terminalis and basal articular diameter of antennal segment III, respectively, BL; length of body, GP; genital plate, HT2; second segment of hind tarsus, SIPH; siphunculi, URS; ultimate rostral segment (segments IV+V).

### DNA extraction, amplification, sequence alignment and data analyses

Total genomic DNA was extracted from one individual of oviparous females using the DNeasy® Blood and Tissue Kit (Qiagen, Inc., Hilden, Germany). The DNA fragments were amplified using AccuPower® PCR PreMix (Bioneer Global Center, Daejeon, South Korea). The partial sequences of the mitochondrial COI gene were amplified using the primer pair LCO1490 (5’–GGTCAACAAATCA TAAAGATATTGG–3’)/HCO2198 (5’–TAAACTTCAGGGTGACCAAAAAATCA–3’; Folmer et al. (1994)). We used the following thermal cycle parameters for 20 microlitre amplification reaction: initial denaturation for 5 min at 94°C, followed by 34 cycles of 1 min at 94°C, 1 min at 45°C and 1 min at 72°C and extension at 72°C for 5 min. PCR products were tested by electrophoresis on a 2% agarose gel and purified using a QIAquick PCR purification kit (Qiagen, Inc., Hilden, Germany), with slight modifications following Choi et al. (2025). Cambodian *S.ralumensis* specimen was successfully amplified and the obtained sequence was deposited in GenBank (https://www.ncbi.nlm.nih.gov/genbank/) under accession number PP976464.

COI raw sequence was examined and contiged using SeqMan II (version 7.0.1, 2007; DNAstar Inc., Madison, WI, USA). Alignments were performed using MAFFT (Katoh et al. 2005, Katoh and Toh 2008) with the default settings in the online sever (ver. 7; https://mafft.cbrc.jp/alignment/software/).

We compared our Cambodian *S.ralumensis* sequence with other reference sequences available in GenBanK. COI sequences of *Schoutedeniaemblica* were searched in the NCBI database. Only three sequences were found; amongst them, two were unpublished and could not be reliably validated. Therefore, only the one published sequence was used in our analysis. For the outgroup, we selected species from other genera within the subfamily Greenideinae, to which *Schoutedenia* also belongs. This is consistent with previous aphid barcoding studies at the genus level, where outgroups are typically chosen from different genera within the same subfamily. Pairwise genetic distances was calculated using MEGA version 11 (Tamura et al. 2021), based on the Kimura 2-parameter (K2P) model (Kimura 1980). A Neighbour-Joining (NJ) tree was constructed with 1,000 bootstrap replicates to assess node support (Saitou and Nei 1987), following the analytical procedures used by Liu et al. (2013) and Choi et al. (2025).

## Taxon treatments

### Schoutedenia ralumensis

Rübsaamen, 1905

6CD7B5B7-92E7-501C-8262-240891777D77

PP976464

#### Materials

- **Type status:** Other material. **Occurrence:** catalogNumber: coll#.111103HR–6; recordedBy: Hwalran Choi; individualCount: 12; sex: female; lifeStage: oviparous females; occurrenceStatus: present; associatedOccurrences: host: *Phyllanthus* sp; associatedSequences: PP976464; occurrenceID: 6577854F-48BB-5AFC-AECB-A3BF2AA5CD7E; **Taxon:** taxonID: 905396; namePublishedInID: Rübsaamen, E.H. 1905. Beiträge zur Kenntnis aussereuropäischer Zooceciden. Marcellia (Rivista Internazionale di Cecidologia) 4: 1–25; namePublishedIn: Rübsaamen, 1905; higherClassification: Animalia; kingdom: Animalia; phylum: Arthropod; class: Insects; order: Hemiptera; family: Aphididae;; kingdom: Animalia; phylum: Arthropoda; class: Insecta; order: Hemiptera; family: Aphididae; genus: Schoutedenia; specificEpithet: ralumensis; taxonRank: species; nomenclaturalCode: ICZN; taxonomicStatus: accepted; taxonRemarks: species; **Location:** continent: Asia; country: Cambodia; countryCode: KH; stateProvince: Koh Kong; locality: Koh Kong Forest Station; decimalLatitude: 11.589; decimalLongitude: 103.354; geodeticDatum: WGS84; coordinateUncertaintyInMeters: 100; **Identification:** identifiedBy: Hwalran Choi; dateIdentified: 16-05-2025; **Event:** samplingProtocol: hand collected; year: 2011; month: 11; day: 11; **Record Level:** type: specimen; modified: 16-05-2025; language: en; rights: Seoul National University; accessRights: not for profit use only; institutionID: NPRI; collectionID: SNU; basisOfRecord: PreservedSpecimen

#### Description

**Oviparous female.**

**Figure 1.**
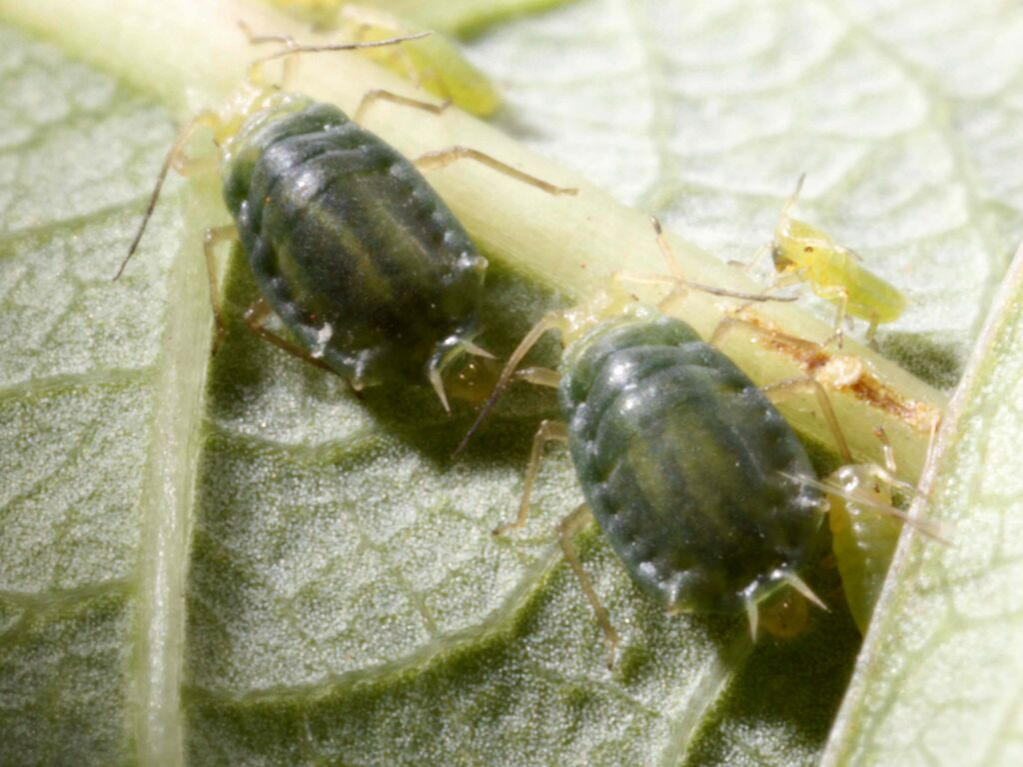
Live photograph of oviparous females of *Schoutedeniaralumensis*.

**Figure 2.**
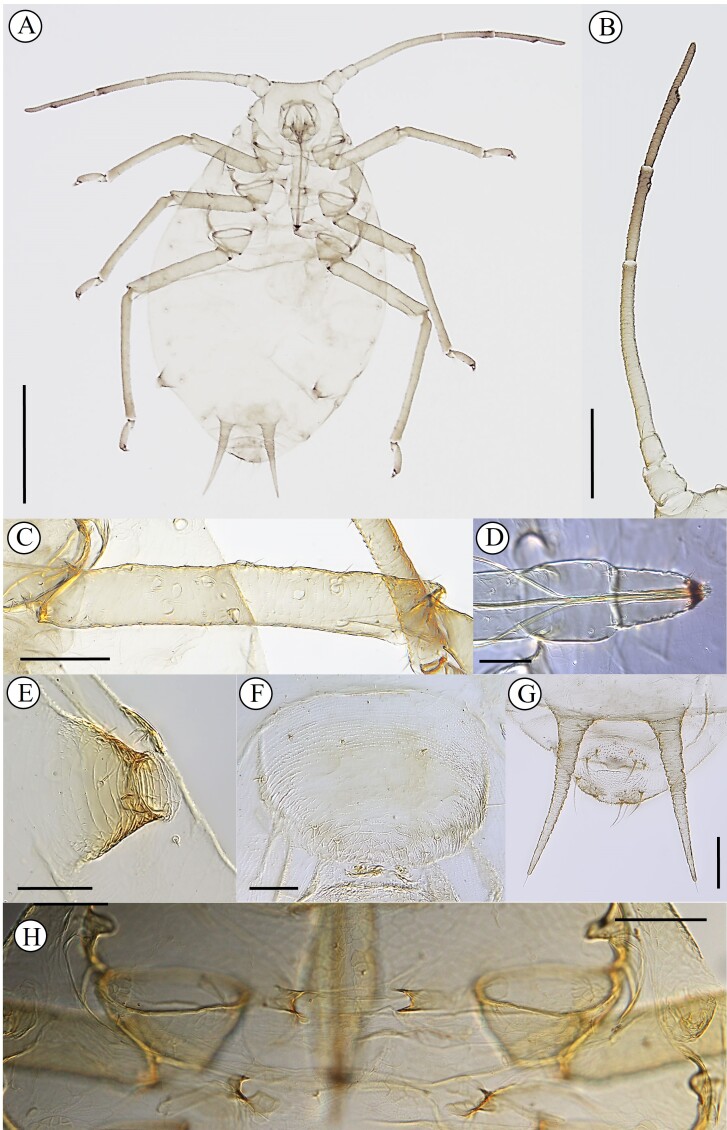
Oviparous females (A–H) of *Schoutedeniaralumensis*. **A** whole body; **B** antenna; **C** hind femur with pseudosensoria; **D** ultimate rostral segment; **E** siphunculus; **F** genital plate; **G** paired spinal processes (spine) on tergite VII; **H** Mesosternal furca. Scale bar indicates: A, 0.5 mm; B, 0.2 mm; C, G, H, 0.1 mm; D, E, F, 0.05 mm.

Colour in life (Fig. 1): Body wholly dark green with vertical stripes on dorsum. Head light green. Ant and legs pale brown. Pigmentation on slide: Head pale like Ant. I-II. (Fig. 2A). Thorax and abdomen pale with dark marginal pigmented sclerites on tergites II–VI (Fig. 2A). Ant wholly pale, except distal part of Ant.IV, Base and PT (Fig. 2B). URS pale (Fig. 2D). Legs pale brown like SIPH, paired spinal processes and cauda (Fig. 2A-F). GP dusky (Fig. 2F).

Morphology. Body oval-shaped, 1.52–1.77 mm long from the most forward point of the frons to end of cauda (Table 1). **Head**: Whole surface of ventrum smooth, that of dorsum granulated; two pairs of setae present on vertex; frons flattened. Antennae 0.44–0.59 times BL, Ant.Ι granulated with 1–2 setae, Ant.II granulated with 2–3 setae, Ant.III without secondary rhinaria with 2–11 setae, longest seta on Ant.III 0.005–0.008 mm and 0.17–0.25 times that of Ant.IIIBD, Ant.IV and between Base and Pt with one primary rhinarium, each. PT 0.56–1.01 times longer than Base. Clypeus without setae, mandibular laminae with 4 setae, rostrum reaching the hind coxae, URS rounded, 0.38–0.68 times longer than PT, 0.26–0.52 times longer than Base with 2–4 median setae. **Thorax**: Prothorax without spinal setae, with one pair of marginal setae. Hind coxae spinulate with 2 acute setae (0.012 mm), trochanter smooth, hind femur with 9–25 round pseudosensoria; first segment of each tarsus smooth with 2 setae (0.026 mm) at apex. Mesosternal furca developed with the arms of the furca separated from each other (Fig. 2H). **Abdomen**: Abdominal segments with faint reticulation, marginal pigmented sclerites distinctly protuberant on tergites II–VI, 2 setae (0.012 mm) on tergite VI between the SIPH, paired spinal processes (spine) on tergite VII, 2 setae on tergite VIII. SIPH truncate conical, 0.56–1.15 times cauda (Fig. 2E), 0.08–0.15 times hind femur, distal half with concentric rings. GP dusky like head, with 2 median setae (0.018 mm). Cauda broadly rounded with 2–5 setae (0.090 mm).

#### Distribution

**Figure 3.**
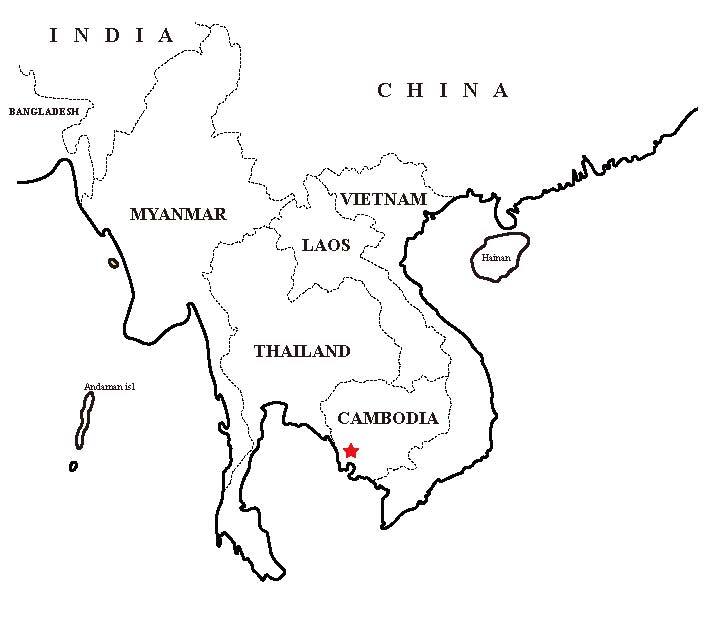
Map showing the collection site of *Schoutedeniaralumensis* in Cambodia (marked with a red star).

Africa, Bangladesh, Cambodia (new record) (Fig. 3), Laos, India, Australia and Papua New Guinea (Favret and Aphid Taxon Community, eds 2025).

#### Notes

The oviparous females of *Schoutedeniaralumensis* are distinguished from other species and forms within the genus by possessing pseudosensoria on the hind femur rather than on the hind tibia — an atypical and potentially diagnostic feature. While the apterous form of this species is typically known to be lemon-yellow or green without stripes when alive, the oviparous form, described here for the first time, displays a green to dark green body with vertical stripes resembling those of a water melon (Fig. 1). The last antennal segment (base and PT) is entirely dark (Fig. 2B). The mesosternal furca is well-developed, with its arms distinctly separated (Fig. 2H). Additionally, the abdominal spiracles are either surrounded by a sclerotised area or raised (Fig. 2A). Other morphological characteristics are consistent with those of viviparous females.

#### Host plant

The Cambodian *S.ralumensis* feeds on the undersides of leaves and stems of *Phyllanthus* species (Phyllanthaceae). Other genera of Phyllanthaceae, such as *Breynia*, *Bridelia*, *Flueggea* and *Glochidion*, have also been recorded as host plants for this species (Favret and Aphid Taxon Community, eds 2025).

## Analysis

### Analysis of the COI barcode

**Figure 4.**
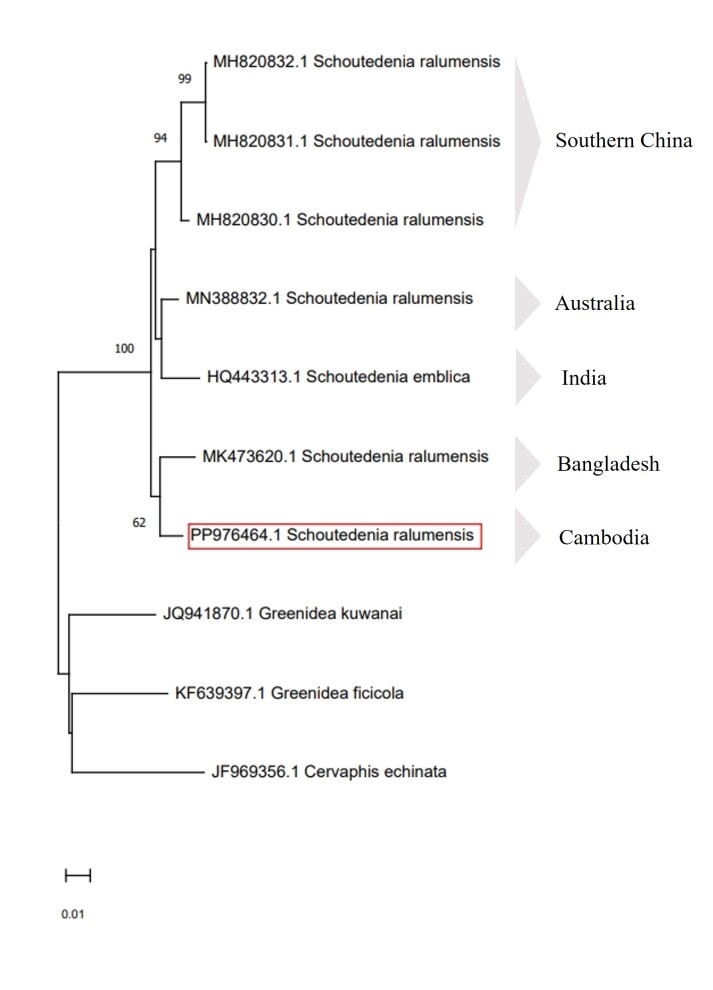
Neighbour–Joining tree, based on Kimura 2–parameter genetic distances. Bootstrap support values are shown at each node. The square box represents *Schoutedeniaralumensis* from Cambodia.

A Neighbour–Joining tree, based on K2P genetic distances, is shown in Fig. 4. Our phylogenetic analysis placed the Cambodian *S.ralumensis* (PP976464.1) within the previously sequenced *S.ralumensis* clade. The K2P genetic distances between the Cambodian specimen and other *S.ralumensis* individuals ranged from 2.17% (vs. Australia, MN388832.1) to 3.39% (vs. China, MH820832.1 and MH820831.1), while the overall intraspecific range within *S.ralumensis* was 0.00% to 4.40% (Table 2). These values are consistent with the broad intraspecific variation previously reported by Liu et al. (2013) for this species (0.00–4.91%).

## Discussion

Species identification in *Schoutedenia* has been complicated by subtle morphological differences and the occurrence of multiple morphs arising from its holocyclic life cycle (Favret and Aphid Taxon Community, eds 2025). Although previous studies (e.g. Liu et al. (2013)) reported relatively high intraspecific COI variation in *S.ralumensis*, similar levels of genetic divergence have also been observed in other widely distributed insect species, suggesting that such variation may fall within the expected range for geographically widespread taxa.

In this study, Cambodian sequence clustered within the known *S.ralumensis* clade, supporting its identification (Fig. 4). More importantly, this record extends the species’ known range to Cambodia and provides the first complete description of the oviparous female, including ecological photographs and DNA barcode data. The discovery of this morph supports the occurrence of a holocyclic life cycle in Southeast Asia and contributes to a clearer understanding of the species’ reproductive biology and regional diversity.

Based on these findings, we propose the synonymisation of *S.emblica* under *S.ralumensis* as a conspecific variant. The morphological differences that were previously used to distinguish the two species — such as the PT and URS ratios — do not appear to represent reliable species-level characters when evaluated alongside genetic data. Instead, these differences likely fall within the range of variation of the *S.ralumensis* species complex. Moreover, the observed genetic divergence appears to be more strongly associated with geographic isolation than with morphological differentiation, further supporting their conspecific status.

## Supplementary Material

XML Treatment for Schoutedenia ralumensis

## Figures and Tables

**Table 1.** Biometric measurements of oviparous females of *Schoutedeniaralumensis* from Cambodia.

Part		Oviparous females (n = 12)	
		Minimun–Maximum	Average
Length (mm)	Body	1.522–1.773	1.681
	Ant.	0.733–0.968	0.862
	Ant.I	0.050–0.082	0.065
	Ant.II	0.043–0.063	0.053
	Ant.III	0.304–0.395	0.353
	Ant.IV	0.144–0.192	0.176
	Base	0.103–0.182	0.144
	PT	0.081–0.127	0.106
	URS	0.038–0.064	0.052
	Hind femur	0.309–0.436	0.377
	Hind tibia	0.423–0.598	0.490
	HT2	0.073–0.120	0.099
	SIPH	0.062–0.097	0.073
	Cauda	0.040–0.071	0.051
	Spine	0.227–0.315	0.272
Ratio of	Ant. / Body	0.438–0.593	0.510
	PT / Base	0.559–1.010	0.743
	PT / Ant.III	0.250–0.357	0.300
	URS / PT	0.376–0.679	0.501
	URS / Base	0.258–0.522	0.371
	SIPH / Body	0.019–0.033	0.024
	SIPH / Ant.III	0.093–0.158	0.117
	SIPH / Hind femur	0.078–0.151	0.109
	SIPH / Cauda	0.559–1.146	0.824
	Setae on Ant.III /Ant.IIIBD	0.171–0.250	0.213
	Setae on tergite VI / Ant.IIIBD	0.176–0.250	0.208
No. of setae on	Mandibular lamina	4	4
	Ant.I	1–2	2
	Ant.II	2–3	2
	Ant.III	2–11	6
	URS	2–4	3
	Abdominal segment VI between SIPH	2	2
	Abdominal segment VIII	2	2
	GP, anterior half	2	2
	GP, posterior half	4–8	6
	Cauda	2–5	4
No. of rhinaria on	Ant.IV	1	1
	PT	1	1

**Table 2.** Pairwise genetic distances between species within the genus *Schoutedenia*, based on the Kimura 2-parameter (K2P) model (Below the diagonal: genetic distances; above the diagonal: standard errors. * indicates a new record of Cambodian *S.ralumensis*).

	MN388832.1 *S.ralumensis*	MH820832.1 *S.ralumensis*	MH820831.1 *S.ralumensis*	MH820830.1 *S.ralumensis*	MK473620.1 *S.ralumensis*	PP976464.1 *S.ralumensis*	HQ443313.1 *S.emblica*
MN388832.1*S.ralumensis* (Australia)		0.80	0.80	0.66	0.71	0.64	0.65
MH820832.1*S.ralumensis* (Southern China)	3.20		0.00	0.49	0.98	0.84	0.91
MH820831.1*S.ralumensis* (Southern China)	3.20	0.00		0.49	0.98	0.84	0.91
MH820830.1*S.ralumensis* (Southern China)	2.23	1.27	1.27		0.90	0.75	0.77
MK473620.1*S.ralumensis* (Bangladesh)	2.62	4.40	4.40	3.81		0.66	0.79
PP976464.1* *S.ralumensis*	2.17	3.39	3.39	2.81	2.39		0.79
HQ443313.1*S.emblica* (India)	2.26	3.81	3.81	2.82	3.46	3.31	
